# Deep Learning of Ultrasound Imaging for Evaluating Ambulatory Function of Individuals with Duchenne Muscular Dystrophy

**DOI:** 10.3390/diagnostics11060963

**Published:** 2021-05-27

**Authors:** Ai-Ho Liao, Jheng-Ru Chen, Shi-Hong Liu, Chun-Hao Lu, Chia-Wei Lin, Jeng-Yi Shieh, Wen-Chin Weng, Po-Hsiang Tsui

**Affiliations:** 1 Graduate Institute of Biomedical Engineering, National Taiwan University of Science and Technology, Taipei 106335, Taiwan; aiho@mail.ntust.edu.tw (A.-H.L.); m10723108@mail.ntust.edu.tw (S.-H.L.); 2 Department of Biomedical Engineering, National Defense Medical Center, Taipei 114201, Taiwan; 3 Department of Medical Imaging and Radiological Sciences, College of Medicine, Chang Gung University, Taoyuan 333323, Taiwan; fierens601@gmail.com (J.-R.C.); rkk36198685@hotmail.com (C.-H.L.); 4 Department of Physical Medicine and Rehabilitation, National Taiwan University Hospital Hsin-Chu Branch, Hsin-Chu 300195, Taiwan; chiaweionly@gmail.com; 5 Department of Physical Medicine and Rehabilitation, National Taiwan University Hospital, Taipei 100225, Taiwan; jyshieh@ntu.edu.tw; 6 Department of Pediatrics, National Taiwan University Hospital, Taipei 100225, Taiwan; 7 Department of Pediatric Neurology, National Taiwan University Children’s Hospital, Taipei 100226, Taiwan; 8 Department of Pediatrics, College of Medicine, National Taiwan University, Taipei 100233, Taiwan; 9 Institute for Radiological Research, Chang Gung University and Chang Gung Memorial Hospital, Linkou, Taoyuan 333323, Taiwan; 10 Division of Pediatric Gastroenterology, Department of Pediatrics, Chang Gung Memorial Hospital at Linkou, Taoyuan 333423, Taiwan

**Keywords:** Duchenne muscular dystrophy, deep learning, ultrasound imaging

## Abstract

Duchenne muscular dystrophy (DMD) results in loss of ambulation and premature death. Ultrasound provides real-time, safe, and cost-effective routine examinations. Deep learning allows the automatic generation of useful features for classification. This study utilized deep learning of ultrasound imaging for classifying patients with DMD based on their ambulatory function. A total of 85 individuals (including ambulatory and nonambulatory subjects) underwent ultrasound examinations of the gastrocnemius for deep learning of image data using LeNet, AlexNet, VGG-16, VGG-16_TL_, VGG-19, and VGG-19_TL_ models (the notation TL indicates fine-tuning pretrained models). Gradient-weighted class activation mapping (Grad-CAM) was used to visualize features recognized by the models. The classification performance was evaluated using the confusion matrix and receiver operating characteristic (ROC) curve analysis. The results show that each deep learning model endows muscle ultrasound imaging with the ability to enable DMD evaluations. The Grad-CAMs indicated that boundary visibility, muscular texture clarity, and posterior shadowing are relevant sonographic features recognized by the models for evaluating ambulatory function. Of the proposed models, VGG-19 provided satisfying classification performance (the area under the ROC curve: 0.98; accuracy: 94.18%) and feature recognition in terms of physical characteristics. Deep learning of muscle ultrasound is a potential strategy for DMD characterization.

## 1. Introduction

Duchenne muscular dystrophy (DMD), an X-linked recessive condition, is a rare genetic disorder caused by the absence of functional dystrophin proteins due to gene mutations [1]. The incidence of DMD is approximately 1 in 5000 male newborns [2]. Affected boys initially exhibit progressive muscle weakness of the lower proximal extremities [3]. The gradual muscle tissue loss and motor function deterioration eventually lead to ambulation loss, with respiratory and cardiac failure at the end stage of the disease [4,5]. Multidisciplinary care and health management are useful strategies to prolong lifespan, improve quality of life, and reduce complications [4]. Several drugs, including corticosteroids, have been conditionally approved for their potential effect on muscle strength and function [6]. Therefore, noninvasive approaches that reliably evaluate DMD are required to support different integrated care plans.

Functional rating scales are commonly used for DMD assessment, including the 6 min walk test and the North Star Ambulatory Assessment [7,8]. These functional measures are limited to only ambulatory measurements and cannot provide quantitative and objective analyses of muscle tissues. Therefore, medical imaging techniques are a crucial diagnostic tool for suspected muscular disorder. Among all imaging modalities, ultrasound imaging offers a real-time, noninvasive, and point-of-care examination to measure muscle size, structure, movement, and function [9]. To characterize tissues, ultrasound imaging biomarkers must be developed to identify neuromuscular disease severity and progression [10]. For example, muscle ultrasound quantification has been proposed by using either mean grayscale measurement of ultrasound B-scan [11,12] or backscattered analysis [11,13] to detect clues associated with muscle pathology. Recent studies have indicated that instantaneous frequency [14], envelope statistics [15], and information certainty [16] of ultrasound backscattered signals are sensitive to variations in tissue microstructures and beneficial for the assessment of DMD severity and ambulatory function.

Notably, quantitative ultrasound analysis requires rigidly fixed settings or a dedicated system for hardware- and software-related reference values during scanning and data acquisition [17]; such an analysis also requires researchers to comprehend the domain knowledge of acoustics so that the clinical outcome can be explained physically [18]. In comparison, deep learning based on a convolutional neural network (CNN) allows us to automatically develop useful features for image classification [19]. A previous study successfully used deep learning for the automated classification of myositis to significantly improve diagnostic accuracy [20]. Deep learning also plays a critical role in computer-aided detection and diagnosis to add the value of muscular ultrasound [21]. This implies that deep learning may be able to endow muscle ultrasound with the ability to evaluate and classify DMD. In addition, seeking relevant ultrasound features through deep learning to fulfill interpretations of the underlying mechanisms and acoustic physics for classifying individuals with ambulatory and nonambulatory DMD is of clinical importance and needs to be explored.

This study investigated the performance of deep learning in ultrasound classification of the ambulatory function of patients with DMD. A total of six CNN models were used (please see the details in the next section), and gradient-weighted class activation mapping (Grad-CAM) was constructed to visualize features recognized by the models. The VGG-19 model provided satisfactory classifications. Grad-CAM revealed that boundary visibility, muscular texture clarity, and posterior shadowing in ultrasound imaging of the gastrocnemius muscle are major features associated with the ambulatory function of patients with DMD.

## 2. Materials and Methods

### 2.1. Study Population

Considering the difficulty in enrolling newly added patients for rare diseases, this study was approved by the Institutional Review Board of National Taiwan University Hospital (Approval code: 201503025RINC; approval date: 30 March 2015) to allow the reuse of the database collected in the previous studies [14,16]. All participants signed informed consent forms, and experiments were conducted according to the approved guidelines. A total of 85 participants aged between 2 and 24 years were recruited. The DMD diagnostics of each patient was confirmed through muscle biopsy or genetic testing. DMD was classified into four stages based on severity: normal control (*n* = 12; no history of weakness or neuromuscular disorders), stage 1 (*n* = 41; ambulatory), stage 2 (*n* = 20; early nonambulatory), and stage 3 (*n* = 12; late nonambulatory). The demographic data of participants and stage definitions were summarized in Table 1.

### 2.2. Ultrasound Data Acquisition

A clinical ultrasound system (t3000; Terason, Burlington, MA, USA) equipped with a linear array transducer (Model 12L5A; Terason) was used for standard-care ultrasound examinations and data acquisition. The central frequency of the transducer was 7 MHz, and the pulse length was 0.7 mm. Through the sagittal scanning approach, the participants underwent scanning of the gastrocnemius muscle, which was recommended as an appropriate location for DMD evaluations [15]. During examinations, the focal length and imaging depth were set as 2 and 4 cm, respectively. Ultrasound scans that excluded acoustic shadowing artifacts and large vessels were performed by a skilled physician to acquire raw image data consisting of 128 backscattered radiofrequency (RF) signals at a sampling rate of 30 MHz. For each raw datum, the absolute values of the Hilbert transform of each backscattered RF signal were calculated to obtain the envelope image, which was then compressed using logarithmic compression to obtain ultrasound B-mode images at a dynamic range of 40 dB.

### 2.3. Data Augmentation

Each B-scan datum was labeled according to DMD diagnosis. The data were divided into training and test sets (the training-to-test ratio in the sample size was at least 3). Considering the limited sample size owing to the rarity of DMD, data augmentation of the training data set was performed through horizontal flipping, random cropping, and translation (lateral direction of the sound beam) for each DMD stage. The amounts of data used for labeling, training, and tests are shown in Table 2.

### 2.4. Deep Learning Approaches

**Figure 1:**
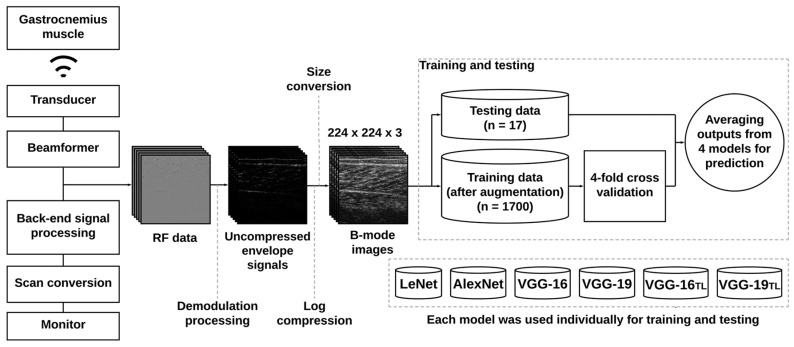
Data training and testing by using the proposed CNN models. Prediction was conducted through averaging the results of four models with four-fold cross-validation.

In this study, LeNet, AlexNet, and VGG models were used as deep learning approaches. LeNet is the classic CNN architecture initially developed for pattern recognition tasks [22]. LeNet consists of two sets of convolutional and average pooling layers, then two fully connected layers, and finally a softmax classifier, providing reductions in run-time complexity for rapid training and testing [22,23]. AlexNet may be treated as an extension model of LeNet, comprising five convolutional layers, three maximum pooling layers, two normalization layers, two fully connected layers, and a softmax layer. Furthermore, AlexNet incorporates rectified linear units as activation functions, which are now the most common choice in CNNs [24]. Compared with AlexNet, a VGG network was developed to provide much deeper networks and much smaller filters in order to learn more complicated image features; this network is popular for medical data analysis [24]. The VGG-16 and VGG-19 models are two common VGG architectures. VGG-16 is composed of five convolutional blocks (including 13 convolutional layers and 3 maximum pooling layers), three fully connected layers, and one softmax layer. VGG-19 consists of 16 convolution layers, 5 maximum pooling layers, 3 fully connected layers, and 1 softmax layer. In addition, the VGG-16 and VGG-19 models pretrained using natural image data sets (ImageNet) were also used for investigations (denoted by VGG-16_TL_ and VGG-19_TL_, respectively). For each CNN architecture, the first two fully connected layers were modified to have 1024 nodes each, and the output layer was adjusted to have two nodes for the binary classification of DMD (ambulatory and nonambulatory subjects). In the training phase, 50 epochs and four-fold cross-validation were used for predicting the test data set. To highlight the relevant ultrasound features of DMD used for predictions, Grad-CAM for each model was obtained using the class-specific gradient information flowing into the final convolutional layer to yield a coarse localization map of the important regions in the image [25]. Data training and tests performed using different models are presented in Figure 1.

### 2.5. Statistical Analysis

To evaluate the performance of each CNN model in classifying participants into ambulatory and nonambulatory groups (normal control and stage 1 DMD versus DMD stages 2 and 3), the sensitivity, specificity, accuracy, precision, and F1-scores were calculated using the confusion matrix. Furthermore, the receiver operating characteristic (ROC) curve analysis was conducted to obtain the area under the ROC curve (AUROC) with a 95% confidence interval. Analyses were performed using MATLAB (R2019a, MathWorks, Natick, MA, USA) and SigmaPlot (version 12.0, Systat Software, Inc., San Jose, CA, USA).

## 3. Results

**Figure 2:**
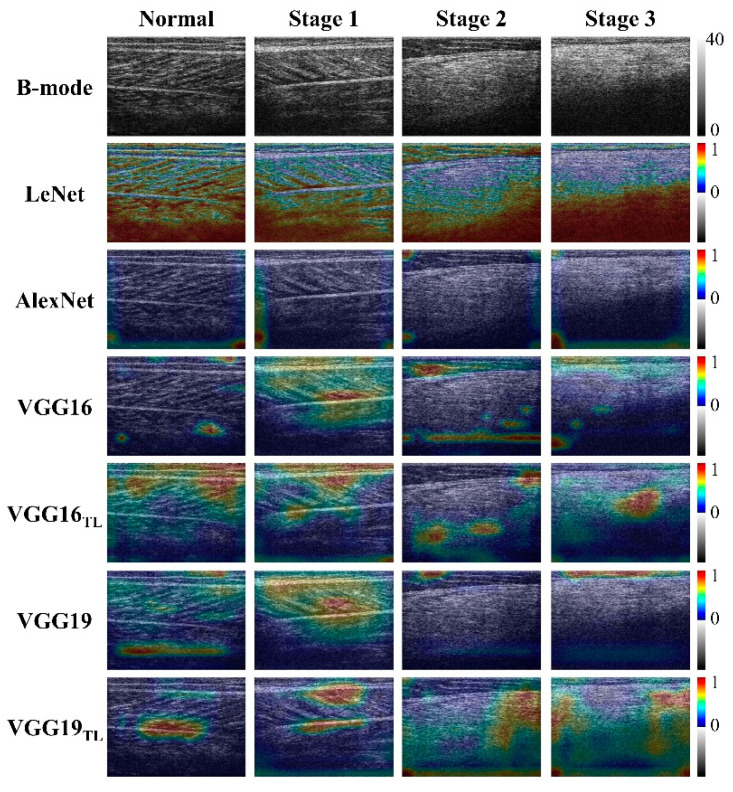
Typical B-mode images and Grad-CAM images of the gastrocnemius muscle corresponding to each stage of DMD. In comparison with B-scan images, the highlighted regions in the Grad-CAM images gradually extend to the inferior boundary of the muscle and the shadowing area in the B-mode image with increasing DMD severity.

The typical ultrasound B-mode images of gastrocnemius muscles in normal controls and patients with different stages of DMD are shown in Figure 2. The brightness of an ultrasound B-scan image increases as the DMD stage increases, indicating that the amplitude of backscattered signals is proportional to DMD severity [16]. The boundaries, structures, and morphological texture were visible and clear in the images for healthy controls and individuals with stage 1 DMD (ambulatory patients); however, the images of gastrocnemius muscles of individuals with DMD stages 2 and 3 (nonambulatory patients) exhibited blurred speckle patterns and hyperechoic regions. In particular, the inferior boundary was not clear, and accompanying shadowing regions were noted. The Grad-CAM images obtained from LeNet, AlexNet, and VGG-based models corresponding to different DMD stages are shown for comparison with ultrasound B-scans. The highlighted regions (the weights of importance) in the Grad-CAM images of healthy controls and individuals with DMD stage 1 appear in the gastrocnemius and are distributed close to inferior and superior boundaries. For Grad-CAM images of individuals with DMD stages 2 and 3, the highlighted regions appear around the inferior boundary and extend to the shadowing area of the B-mode image.

**Figure 3:**
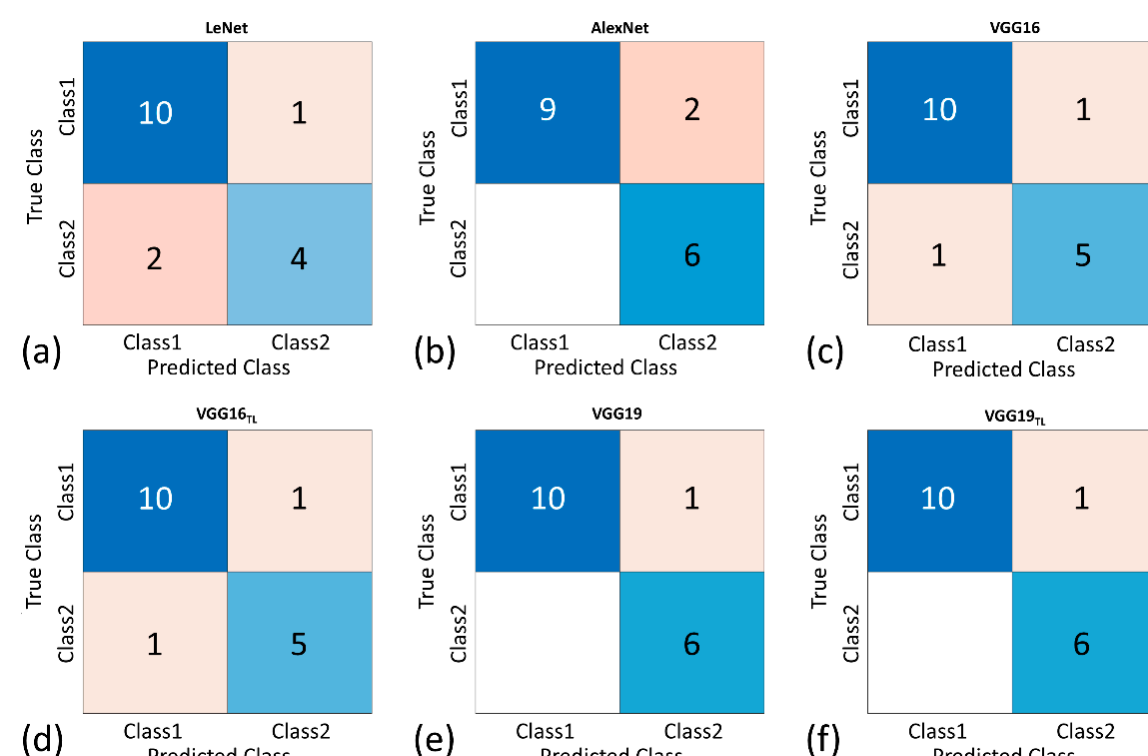
The confusion matrix of predicting ambulatory function of the patients with DMD for each model (class 1: *n* = 11 for normal control and stage 1; class 2: *n* = 6 for stages 2 and 3). (**a**) LeNet; (**b**) AlexNet; (**c**) VGG-16; (**d**) VGG-16_TL_; (**e**) VGG-19; and (**f**) VGG-19_TL_. VGG-19 provided the highest true positive and true negative rates in the test dataset.

**Figure 4:**
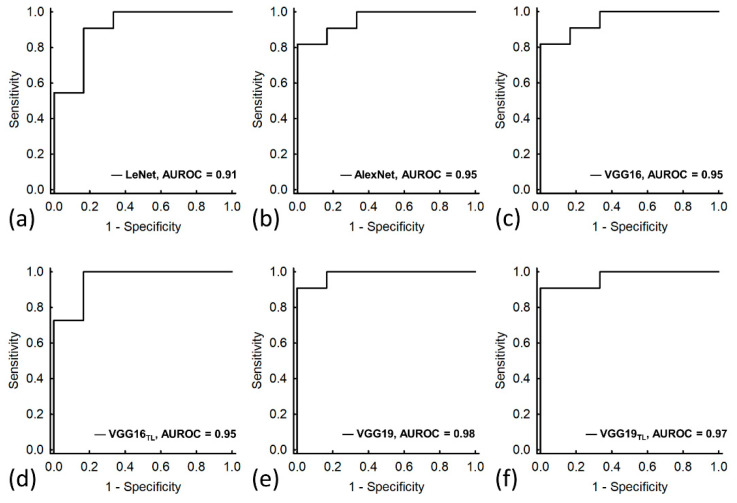
ROC curves when using the proposed model to differentiate the ambulatory and nonambulatory patients. (**a**) LeNet; (**b**) AlexNet; (**c**) VGG-16; (**d**) VGG-16_TL_; (**e**) VGG-19; and (**f**) VGG-19_TL_. VGG-19 as well as its pretrained version offered higher AUROCs compared with other proposed models.

The confusion matrix of predicting ambulatory function of the patients with DMD for each model is shown in Figure 3. Among the proposed models, VGG-19 provided the highest true positive and true negative rates in the test dataset (accuracy: 94.18%; precision: 85.71%; sensitivity: 100%; specificity: 90.91%; F1-score: 0.92). VGG-19 also had the highest diagnostic performance (AUROC: 0.98) in the functional classification of DMD, as shown in Figure 4 and Table 3.

## 4. Discussion

### 4.1. The Significance of This Study

This is the first study to explore the feasibility of using deep learning of ultrasound imaging in predicting the ambulatory status of patients with DMD. Both the basic architectures and pretrained CNN models used for validating the proposed research idea are well-developed deep learning solutions that benefit reduction in the technical barrier in practical uses to accelerate clinical applications. The results obtained from the clinical data set of DMD show that deep learning endowed ultrasound imaging with the ability to evaluate DMD and perform feature recognition in terms of physical characteristics. Comparatively, VGG-19 offered satisfactory performance and confusion matrix data in detecting changes in the ambulatory function of patients with DMD.

### 4.2. Considerations on Ultrasound Evaluations of DMD

The progression of DMD involves two critical periods, namely when dystrophia occurs and when patients lose their ambulation. Free-acting capability is an important index for life quality, mental health, and disease management of patients, and therefore, prolonging ambulatory function is the major aim of DMD treatment [26]. Moreover, evaluation and prediction of change in ambulatory function are helpful for individual treatment planning, including corticosteroid adjustment and rehabilitation in multidisciplinary care, which are imperative for alleviating muscle atrophy, skeletal deformities, and motor function deterioration [27,28]. Clinically, observations on strength loss (e.g., hip extension and ankle dorsiflexion) are typically used as the primary evaluation of ambulation loss in patients with DMD [29]. To compensate for muscle weakness, patients with DMD naturally develop compensatory movements; however, before compensatory movements are used, structural changes in muscle may exist already to gradually influence muscular function and the corresponding image features. Evidently, the gastrocnemius muscle is more sensitive to reflecting progressive changes in the muscle architecture in ambulatory boys with DMD [15]. A previous study also suggested that the gastrocnemius is the earliest affected muscle and could be useful for disease monitoring in ambulatory boys [30]. In other words, ultrasound image patterns of the gastrocnemius muscle may be critical clues in ultrasound assessment of ambulatory function in DMD patients.

### 4.3. Physical Interpretations of Deep Learning in Ultrasound Imaging of DMD

Deep learning should be used carefully in dealing with medical problems because an accurate classification of medical data is not all that is required [31]. Identifying physical characteristics that are beneficial for clinical interpretations of the disease is essential for further understanding related mechanisms. A previous study suggested opening the black box of artificial intelligence to extend domain knowledge [32]. Grad-CAM allows CNN-based models to be more transparent by visualizing input areas with details that are useful for prediction. By using the Grad-CAM technique, we explained the image pattern recognized by CNN models and better comprehended how these CNN models characterize DMD. The Grad-CAM results indicate that boundary visibility, muscular texture clarity, and posterior shadowing are highlighted features in ultrasound imaging of the gastrocnemius recognized by the models for evaluating ambulatory function, as shown in the Results section. Recall that the echo intensity of ultrasound B-scans for normal muscles is relatively low, and dystrophic muscles behave similarly to hyperechoic tissue due to intramuscular fat infiltration and fibrosis [33,34]. In addition, fatty infiltration increases the strength of backscattered signals, resulting in decreased ultrasound beam energy for tissue penetration (i.e., acoustic attenuation) [35,36]. In this circumstance, the speckle pattern was blurred and hyperechoic, and the shadowing effect occurred under the inferior boundary of muscle when DMD severity increased. Owing to advancements in deep learning, the above acoustically structural features can be now recognized by deep learning to assist in the physical interpretation of imaging findings when sonographic examinations of DMD are performed for quantitative classification. Notably, Grad-CAM images obtained from different models performed differently in visualizing each sonographic feature. As shown in Figure 2, LeNet and AlexNet were relatively sensitive to the shadowing effect caused by acoustic attenuation; VGG-based models tended to recognize features related to boundary visibility and muscular texture clarity. Because the current results show that VGG-19 outperformed the other proposed models, boundary visibility and muscular texture clarity may be clues that are more relevant to ambulatory function for DMD patients.

### 4.4. Comparisons with the Proposed Models

Notably, obtaining comprehensively annotated medical data on patients with DMD is challenging. Transfer learning (i.e., fine-turning CNN models pretrained on a large annotation data set) is conventionally believed to be a useful method for training deeper networks without overfitting and improving performance and training time [37]. However, we found that pretrained models (VGG-16_TH_, VGG-19_TH_) did not significantly outperform those without transfer learning (LeNet, AlexNet, VGG-16, and VGG-19) in classifying the ambulation status of individuals. Probably due to the nature of ultrasound images of muscle tissues, the transfer learning technique based on natural image data sets could not provide the deep learning model with the ability to recognize changes in sonographic features. It is a challenging problem to improve performance by transferring knowledge from another domain to the medical ultrasound domain [37]. Comparatively, using a much deeper network (VGG-19) was more useful in enhancing the performance of deep learning in learning DMD-related image features, as shown in the comparisons in Table 3. This may be due to the fact that shallow networks are good at memorization, but do not perform well for generalization. Multiple layers are beneficial for learning features at various levels of abstraction, achieving better image pattern characterization and classification [38]. However, using much deeper networks may be unable to promise state-of-the-art results for all medical applications; for example, increasing complexity and depth of networks for the classification of chest radiographs is not necessarily a requirement to achieve more outstanding performance [39].

### 4.5. Limitations of This Study

This study has some limitations. First, the sample size was small because of the rarity of DMD. A large sample size is useful for data augmentation and necessary for further investigations of multiclass classifications. Second, the image data used in this study were obtained through reconstructions of raw RF signals (without any signal and image processing). However, clinical ultrasound systems allow adjustment of imaging parameters and settings, making the image quality system-dependent. The effects of system characteristics on deep learning–based classification should be further clarified. Furthermore, a cross-platform investigation should be considered in future research.

## 5. Conclusions

This study has demonstrated the value of deep learning in muscle ultrasound evaluations of individuals with DMD by clinical data analysis. The results indicate that the basic architectures and pretrained CNN models performed well in differentiating individuals with ambulatory and nonambulatory DMD. Boundary visibility, muscular texture clarity, and posterior shadowing in ultrasound imaging of the gastrocnemius were recognized by the models as major features associated with the ambulatory function of patients with DMD. Compared with the other proposed models, VGG-19 outperformed in classifying ambulatory function and recognizing sonographic features of DMD. The current clinical findings indicate that deep learning endows ultrasound imaging with the diagnostic ability to characterize DMD by providing interpretations of the underlying imaging physics. In the future, deep learning of muscle ultrasound may be a potential strategy to benefit the clinical evaluation and monitoring of disease progression for patients with DMD.

## Figures and Tables

**Table 1:** Patient demographic data and DMD stage definitions. DMD was diagnosed through muscle biopsy or genetic testing and classified into four stages according to clinical symptoms.

Stage	Clinical Symptoms	Age (Years) (Range)	Number of Subjects
Normal	No weakness: No neuromuscular disorders or weakness	10.75 ± 4.59 (3–18)	12
Stage 1	Presymptomatic: Subtle symptoms of delayed walking or delayed speech (but often unnoticed). Early ambulatory: Showing a Gowers’ sign (patients need to support themselves with hands to get up from the floor), waddling type walking (gait), and walking on their toes. Late ambulatory: Walking becomes increasingly difficult (labored gait) and climbing stairs and getting up from the floor are more problematic.	7.92 ± 2.33 (2–13)	41
Stage 2	Early non-ambulatory: Patients start to need to use a wheelchair; they may be able to wheel the chair themselves and typically their postures can be maintained even scoliosis is possible.	12.68 ± 2.05 (9–16)	20
Stage 3	Late non-ambulatory: Upper limb function and maintenance of good posture are increasingly difficult, and complications are more likely.	17.08 ± 2.90 (13–24)	12

**Table 2:** Sample size and amount of data for labeling, training, and tests of ultrasound images for patients with DMD.

Subjects	Number of Subjects	Number of Subjects (Training, Test)	Amount of Training Data (after Augmentation)
Normal control	12	(10, 2)	250
Stage 1	41	(32, 9)	800
Stage 2	20	(16, 4)	400
Stage 3	12	(10, 2)	250

**Table 3:** Performance metrics for differentiating ambulatory and nonambulatory patients through deep learning of ultrasound imaging data using the proposed models. Compared with LeNet, AlexNet, VGG-16, and VGG-16_TH_, VGG-19 as well as its pretrained version offered better performance in terms of confusion matrix data.

Model	LeNet	AlexNet	VGG-16	VGG-16_TL_	VGG-19	VGG-19_TL_
Accuracy, %	82.35	88.24	88.24	88.24	94.18	94.18
Precision, %	80.00	75.00	83.33	83.33	85.71	85.71
Sensitivity, %	66.67	100.00	78.82	78.82	100.00	100.00
Specificity, %	90.91	81.82	90.91	90.91	90.91	90.91
F1-score	0.73	0.86	0.83	0.83	0.92	0.92
AUROC (95% CI)	0.91 (0.75–1.00)	0.95 (0.87–1.00)	0.95 (0.87–1.00)	0.95 (0.85–1.00)	0.98 (0.94–1.00)	0.97 (0.90–1.00)

AUROC area under the receiver operating characteristic (ROC) curve, CI: confidence interval.

## Data Availability

The data presented in this study are available on request by contacting the corresponding author.
